# *Thespesia* *lampas* mediated green synthesis of silver and gold nanoparticles for enhanced biological applications

**DOI:** 10.3389/fmicb.2023.1324111

**Published:** 2024-01-05

**Authors:** Sunayana Nath, Ritis Kumar Shyanti, Rana Pratap Singh, Manoj Mishra, Bhawana Pathak

**Affiliations:** ^1^School of Environment and Sustainable Development, Central University of Gujarat, Gandhinagar, Gujarat, India; ^2^School of Life Sciences, Jawaharlal Nehru University, New Delhi, India; ^3^Cancer Biology Research and Training Program, Department of Biological Sciences, Alabama State University, Montgomery, AL, United States

**Keywords:** medicinal plant, silver and gold nanoparticles, green synthesis, antibacterial activity, anticancer activity, spheroid, DNA protection activity, *Thespesia lampas*

## Abstract

The present study investigated the synthesis and biological applications of green, economical, and multifunctional silver and gold nanoparticles (TSAgNPs and TSAuNPs) using the ethnomedical important medicinal plant *Thespesia lampas* for biological activities. Relatively higher levels of antioxidant components were measured in *T. lampas* compared to the well-known *Adhatoda vasica*, and *Diplocyclos palmatus* suggested the potential of *T. lampas* for the study. Synthesized TSAgNPs and TSAuNPs were characterized through UV–Vis, XRD, SEM-EDS, HR-TEM, SAED, and FTIR techniques. SEM revealed that TSAgNPs and TSAuNPs were predominantly spherical in shape with 19 ± 7.3 and 43 ± 6.3 nm crystal sizes. The sizes of TSAgNPs and TSAuNPs were found to be12 ± 4.8 and 45 ± 2.9 nm, respectively, according to TEM measurements. The FTIR and phytochemical analyses revealed that the polyphenols and proteins present in *T. lampas* may act as bio-reducing and stabilizing agents for the synthesis. Synthesized NPs exhibited enhanced scavenging properties for ABTS and DPPH radicals. TSAgNPs and TSAuNPs were able to protect DNA nicking up to 13.48% and 15.38%, respectively, from oxidative stress. TSAgNPs possessed efficient antibacterial activities in a concentration-dependent manner against human pathogenic bacteria, such as *E. coli*, *B. subtilis*, *P. vulgaris,* and *S. typhi*. Furthermore, TSAgNPs and TSAuNPs showed significant cytotoxicity against FaDu HNSCC grown in 2D at 50 and 100 μg mL^−1^. Tumor inhibitory effects on FaDu-derived spheroid were significant for TSAgNPs > TSAuNPs at 100 μg mL^−1^ in 3D conditions. Dead cells were highest largely for TSAgNPs (76.65% ± 1.76%), while TSAuNPs were non-significant, and Saq was ineffectively compared with the control. However, the diameter of the spheroid drastically reduced for TSAgNPs (3.94 folds) followed by TSAuNPs (2.58 folds), Saq (1.94 folds), and cisplatin (1.83 folds) at 100 μg mL^−1^. The findings of the study suggested the bio-competence of TSAgNPs and TSAuNPs as multi-responsive agents for antioxidants, DNA protection, antibacterial, and anti-tumor activities to provide a better comprehension of the role of phytogenic nanoparticles in healthcare systems.

## 1 Introduction

Metal nanoparticles (NPs), such as silver and gold, have gained researchers’ attention as potential nanoproducts that find imperative applications in biomedicine (Hawsawi et al., 2023; Nath et al., 2023; Sukri et al., 2023). On the ground of medical applications of silver and gold nanoparticles, there are various reports on antibacterial investigations (Rasheed et al., 2017; Nath et al., 2020; Wypij et al., 2021; Singh and Mijakovic, 2022; Raja et al., 2023), antifungal activities (AlMasoud et al., 2020; Leyu et al., 2023), anticancer studies (Gomathi et al., 2020; Kumari et al., 2020; Nath et al., 2020; Anadozie et al., 2023; Moosavy et al., 2023; Raja et al., 2023), antioxidant evaluations (Rasheed et al., 2017; Leyu et al., 2023; Moosavy et al., 2023), wound healing studies (Aldakheel et al., 2023), anthelmintic activity (Majumdar and Kar, 2023), and anti-inflammatory and analgesic activities (Ahmad et al., 2015). On the other hand, the chemicals employed in the fabrication of nanomaterials are expensive but also toxic and hazardous to human life and the environment (Dehvari and Ghahghaei, 2018; Rahimi and Doostmohammadi, 2019; Altammar, 2023). The byproducts produced during the nanomaterial synthesis reactions eventually lead to various biological risks that limit their application in biomedical and clinical fields (Khan et al., 2021). On the other hand, the green chemistry approach of utilizing biological systems to synthesize nanoparticles is a non-toxic, clean, biocompatible, and environment-friendly technique (Liaqat et al., 2022). Botanical extract-mediated synthesis of metal nanoparticles has offered a biocompatible and economical fabrication (Nath et al., 2020; Singh and Mijakovic, 2022). Using crude extracts of plant parts to fabricate NPs suggests a better option for high-yield production. Fruit extract of *Couroupita guianensis* and *Punica granatum* (Sathishkumar et al., 2016; Sukri et al., 2023), leaf of *Lawsonia inermis* (Ajitha et al., 2016), peel of *Nephelium lappaceum* (Kumar et al., 2015), stem of *Tinospora cordifolia* (Nath et al., 2023), roots of *Erythrina indica* (Sre et al., 2015), and leaf of *Carica papaya* (Singh et al., 2021) have been cited for reducing and stabilizing green NPs. Plants owe a diverse range of phytochemicals, metabolites, and antioxidant compounds, including polyphenols, lignin, polysaccharides, and cellulose, that provide excellent bio-reductants and bio-stabilizers (Susanti et al., 2022; Kulkarni et al., 2023). These active herbal components may act separately or synergistically to prevent the agglomeration of NPs by forming a bio-layer around the NPs (Mishra et al., 2013b).

*Thespesia lampas* is not a well-known ethnomedical medicinal plant. The plant has been reported for various therapeutic properties including hepatoprotective (Ambrose et al., 2012), antioxidant (Sangameswaran et al., 2009), anthelmintic (Kosalge and Fursule, 2009), anti-diabetic (Jayakar and Sangameswaran, 2008), and antimicrobial studies (Valsaraj et al., 1997). The root, stem, and leaves have been reported for anti-inflammatory, anthelmintic acidity, bleeding nose, bronchitis, carbuncle, cough, dysentery, fever, gonorrhea, sunstroke, and urinary complaints (Adhikari et al., 2007). Recently, the stem part has been explored for its cellulose fibers (Chumbhale and Upasani, 2012; Reddy et al., 2014; Ashok et al., 2015, 2019) and derived silver NPs (Ashok et al., 2018). The therapeutic potential and convenient availability of the stem part made it a suitable choice to be included in the study. Therefore, the presented study is a systematic effort to investigate (i) the phytochemical profile of *T. lampas* and compare it with two medicinal plants of repute, namely, *Adhatoda vasica* (Gantait and Panigrahi, 2018) and *Diplocyclos palmatus* (Packer et al., *2012*); (ii) the synthesis of *T. lampas* stem-mediated silver and gold nanoparticles (TSAgNPs and TSAuNPs); (iii) the multi-responsive functions of TSAgNPs and TSAuNPs for radical scavenging activity, DNA protective potential, and broad-spectrum antibacterial properties; (iv) cytotoxicity against the FaDu head and neck squamous cell carcinoma cells (HNSCCs) in 2D and 3D conditions using FaDu-derived spheroid. The study is the first report on the biological potential of *T. lampas*-encapsulated TsAgNPs and TSAuNPs.

## 2 Materials and methods

All chemicals used to synthesize NPs, phytochemical estimation, cytotoxic, antioxidant_,_ and antibacterial studies were purchased from HiMedia (Mumbai, India). Cell culture media and fetal bovine serum were obtained from Invitrogen Life Technologies (Grand Island, NY), and pUC19 DNA was obtained from Sigma–Aldrich (St Louis, MO). Calcein AM, Ethidium Bromide, and Hoechst 33342 dyes were procured from Life Technologies (Thermo Fisher Scientific, Waltham, MA). Extracts and NPs were prepared using Millipore Milli-Q water (Merck Millipore, Massachusetts, United States). All chemicals were of analytical grade.

### 2.1 Sample collection and identification

Stems of *A. vasica*, *D. palmatus,* and *T. lampas* were collected from Dhareshwar Mount in Vijayanagar Forest, North Gujarat, India. The voucher number for taxonomic identification (*A. vasica* SN-01/BSJO, *D. palmatus* SN-06/BSJO, and *T. lampas* SN-13/BSJO) was provided by the Arid Zone Regional Center, Botanical Survey of India, Government of India.

### 2.2 Preparation of aqueous and hydromethanolic extracts

Approximately 10 g shade-dried powder of *T. lampas* stem was extracted in 100 mL water at 60°C for 30 min, centrifuged, and the supernatant was collected. The exhausted pellet was re-extracted, and combined supernatants (5%) of stem aqueous extract (Saq) were collected. Hydromethanolic extracts of the dried stems (500 mg in 80% methanol) of all three plants were prepared according to Nath et al. (2017).

### 2.3 Determination of polyphenols and antioxidant activity of *Adhatoda vasica*, *Diplocyclos palmatus,* and *Thespesia lampas*

The total phenolic content (TPC) of the extracts was assessed according to the Folin–Ciocalteau method (Cai et al., 2004). In brief, 0.25 mL of hydromethanolic extracts was added to 0.25 mL of 2 N Folin–Ciocalteu reagent and was neutralized by 7% (w/v) sodium carbonate and kept in the dark at room temperature (RT) for 90 min. The absorbance of the resulting blue color was measured at 765 nm using a multimode plate reader (Synergy H1 Hybrid Multi-Mode Microplate Reader). The results are expressed in mg gallic acid equivalent per g dry weight (GAE/g DW) basis.

The total flavonoid content (TFC) of samples was determined according to the aluminum chloride method (Nath et al., 2017). In total, 500 μL of hydromethanolic extracts was added to AlCl_3_ (500 μL, 10% w/v) and potassium acetate (100 μL, 1.0 M). The mixtures were incubated at 22°C ± 1°C for 30 min, and the absorbance was measured at 415 nm. Data are expressed in mg quercetin equivalent (QE/g DW).

Diluted ABTS solution (absorbance of 0.700) was added to 100 μL of hydromethanolic extracts (0.5–2 mg mL^−1^) and mixed thoroughly (Cai et al., 2004). The reaction mixture was allowed to stand for 6 min in the dark, and the absorbance was measured at 734 nm. The radical scavenging activity (RSA) % was calculated as:


(1) $$RSA\%=\frac{A_{0}-A_{1}}{A_{0}}X\;100$$

where A_0_ is the absorbance of the control (without test sample), and A_1_ is the absorbance of the reaction mixture (with test sample). Trolox (0.03 to 0.2 mg mL^−1^) was used as a positive control.

DPPH RSA % was measured as described by Blois (1958). Overall, 100 μL of hydromethanolic extracts (0.5–2 mg mL^−1^) was mixed with 2 mL of DPPH. The absorbance of the reaction mixture was measured at 517 nm. Ascorbic acid (0.02 to 2 mg mL^−1^) was used as a positive control. The percentage of DPPH decolorization of the sample was calculated according to Equation 1.

Total antioxidant capacity (TAC) was measured according to Prieto et al. (1999). Hydromethanolic extract aliquots were added to 1 mL of reagent solution containing 0.3 N sulfuric acid, 4 mM ammonium molybdate, and 28 mM sodium phosphate. Tubes were placed at 100°C for 90 min and cooled to RT, and the absorbance was noted at 695 nm. The result is expressed as μM ascorbic acid equivalent (AAE/g DW).

### 2.4 Synthesis of TSAgNPs and TSAuNPs

To synthesize TSAgNPs and TSAuNPs, 10 mL of saq (5%, pH 6) was added drop by drop into two separate flasks containing 90 mL 2 mM AgNO_3_ and 90 mL 2 mM HAuCl_4_ solutions in the mixing ratio of 1:9, respectively. Both flasks were continuously stirred at 400 rpm for 24 h at RT. After that, colloidal solutions were centrifuged at 14,000 rpm at 4°C for 20 min and washed with ethanol and Milli-Q water to harvest purified TSAgNPs and TSAuNPs (Ramteke C. et al., 2013). NPs were air-dried, crushed to powder, and stored in the dark.

### 2.5 Physical characterization

The absorption spectra of NPs were monitored through UV–vis spectrophotometer (Analytical, 2060+). X-ray diffraction (XRD) pattern was recorded using X-ray diffractometer (X’Pert Pro, PANalytical, BV) operated at 40 kV, 30 mA, CuKα (k = 1.5406 Å), K-bet filter in the 2θ range of 10°–80° with a continuous scanning speed of 10°/min. Surface morphology was analyzed using field-emission scanning electron microscopy (FE-SEM; Bruker NANO NOVA 450); the Energy dispersive X-ray spectrum (EDS) was recorded at 20 kV (Bruker, Germany). Size and surface morphology were analyzed using high-resolution transmission electron microscopy (HR-TEM; JEOL model, JEM-2000FX) and selected area electron diffraction (SAED). Fourier transform infrared (FTIR; KBr pellet method) was used to show the functional groups in the range of 500–4,000 cm^−1^ (Perkin Elmer, SP-65).

### 2.6 Estimation of polyphenols before and after synthesis reaction

Polyphenol levels (TPC and TFC) before the synthesis reaction, i.e., Saq, and after the synthesis reaction (solution left after harvesting NPs) were estimated as described in section 2.3.

### 2.7 Extraction and estimation of protein before and after synthesis reaction

Determination of protein content was performed according to the Bradford method (Bradford, 1976). Approximately 50 mg of sample was mixed with 1 mL of extraction buffer containing 1 M Tris–HCl pH 6.8, 50% glycerol, 25% beta-mercaptoethanol, 10% SDS, and 1% bromophenol blue. The mixture was denatured by placing at 100°C for 10 min, followed by 2 min of vortex and centrifugation at 5,600 rpm for 8 min. The supernatant (10 μL) was added to 5 mL Coomassie brilliant blue dye, and the absorbance was measured at 630 nm. The value is expressed as mg BSA/g DW.

Furthermore, the proteins involved in the synthesis of TSAgNPs and TSAuNPs were investigated through SDS-polyacrylamide gel electrophoresis analysis as described previously (Mishra et al., 2013a). Overall, 20–25 μg of protein was taken as a loading sample for separation on 10% SDS-PAGE and fixed in 12.5% trichloroacetic acid for 1 h at RT. The gel was stained with Coomassie brilliant blue. Furthermore, the gel was washed to distain the dye. After proper distaining, an image was captured to show the ladder and protein bands in the gel.

### 2.8 Antioxidant activity of TSAgNPs and TSAuNPs

ABTS and DPPH RSA % of Saq, TSAgNPs, and TSAuNPs were calculated as described in section 2.3.

### 2.9 DNA damage protective potential

DNA damage protective assay was performed using a Fenton reagent following the method proposed by Lee et al. (2002). In total, 50 μL of the reaction mixture contained 2 μL pUC19 DNA, volume of 30 mM H_2_O_2_, 80 mM FeCl_3_, 50 mM ascorbic acid in PBS, and 10 μL of 100 μg mL^−1^ Saq, volume of 20 μg mL^−1^ of TSAgNPs/TSAuNPs. The tubes were incubated at 37°C for 30 min. The mixture was then loaded with 2 μL of bromophenol dye onto 0.8% agarose gel and ran for 1 h at 90 V with 0.5X TBE buffer. DNA bands were stained with ethidium bromide and captured using the Syngene gel documentation system.

### 2.10 Evaluation of antibacterial activity

Antibacterial assay was performed using the agar well diffusion method (Sre et al., 2015). In total, 100 μL inoculum of human pathogenic bacteria, such as *E. coli*, *B. subtilis*, *P. vulgaris*, and *S. typhi,* at their log phase containing density of approximately 1.5 × 10^8^ cfu mL^−1^ was poured onto a solidified agar plate and gently spread with the help of sterile cotton swab. Wells were punched as per requirement on the agar plate using a sterile metal borer with a diameter of 0.7 mm. In total, 50 μL of test samples (0.5–50 μg mL^−1^ TSAgNPs and TSAuNPS for concentration-dependent study, 20 μg mL^−1^ TSAgNPs and 20 μg mL^−1^ TSAuNPs for comparative study, 5% Saq, 2 mM AgNO_3_, and 2 mM HAuCl_4_) was injected into the wells and incubated at 37°C for 24 h. At the end of the incubation period, plates were observed to record the zone of inhibition (ZOI).

### 2.11 *In vitro* cytotoxic assay

Extracts and NPs were analyzed for their cytotoxic activities against the FaDu HNSCC growing at monolayer (2D condition) using the MTT method as previously described (Shyanti et al., 2017). Cells were seeded at 1 × 10^4^ cells per well in 96-well plates in EMEM medium supplemented with 10% FBS. Cultured cells were treated (in triplicates) with samples (Saq, TSAgNPs, and TSAuNPs) at 50 and 100 μg mL^−1^ concentrations in 1% DMSO. Plates were incubated in 5% CO_2_ at 37°C for 24 h. After incubation, 20 μL of MTT (5 mg mL^−1^) solution was added to each well and incubated for the next 4 h. After the time period, 100 μL of DMSO was added to each well, and the absorbance was measured at 570 nm in the multimode reader. The proliferation percentage of viable cancer cells was calculated relative to untreated DMSO as a control.

### 2.12 *In vitro* anti-tumor spheroid multi-stain assay

FaDu cells were plated (100 μL) at 1,000 cells per well into a Corning 96-well ultralow attachment plate in a DMEM F12 medium supplemented with EGF, b-FGF, wnt, noggin, R-spondin, and B27. The plate was kept inside a 5% CO_2_ incubator at 37°C. Spheroid formation was observed on day 3. On day 7, spheroids were treated with control (DMSO), positive control-cisplatin (10 μg mL^−1^), saq (100 μg mL^−1^), TSAgNPs (100 μg mL^−1^), and TSAuNPs (100 μg mL^−1^) and subsequently observed for morphological changes and spheroid viability. After 72 h (on day 10), Hoechst 33342 (1 μM), calcein AM (2 μM), and EtBr (1 μM) cocktail in 1× PBS were overlaid on each well having spheroids and incubated for 15–20 min. Calcein AM-stained cells were live cells observed as green (FITC channel), EtBr-stained cells were dead cells observed as red (TRITC channel), and Hoechst 33342-stained cells were observed as blue (UV channel; Pandey et al., 2022).

### 2.13 Statistical analysis

All experiments were performed with three technical replicates. The results are represented as mean value ± standard deviation. The significance of the difference was analyzed through one-way ANOVA following Tukey’s test (*p* < 0.05) with GraphPad Prism version 6.01 (La Jolla, CA).

## 3 Results and discussion

### 3.1 Polyphenols and antioxidant potential of *Thespesia lampas*, *Adhatoda vasica*, and *Diplocyclos palmatus*

**Figure 1:**
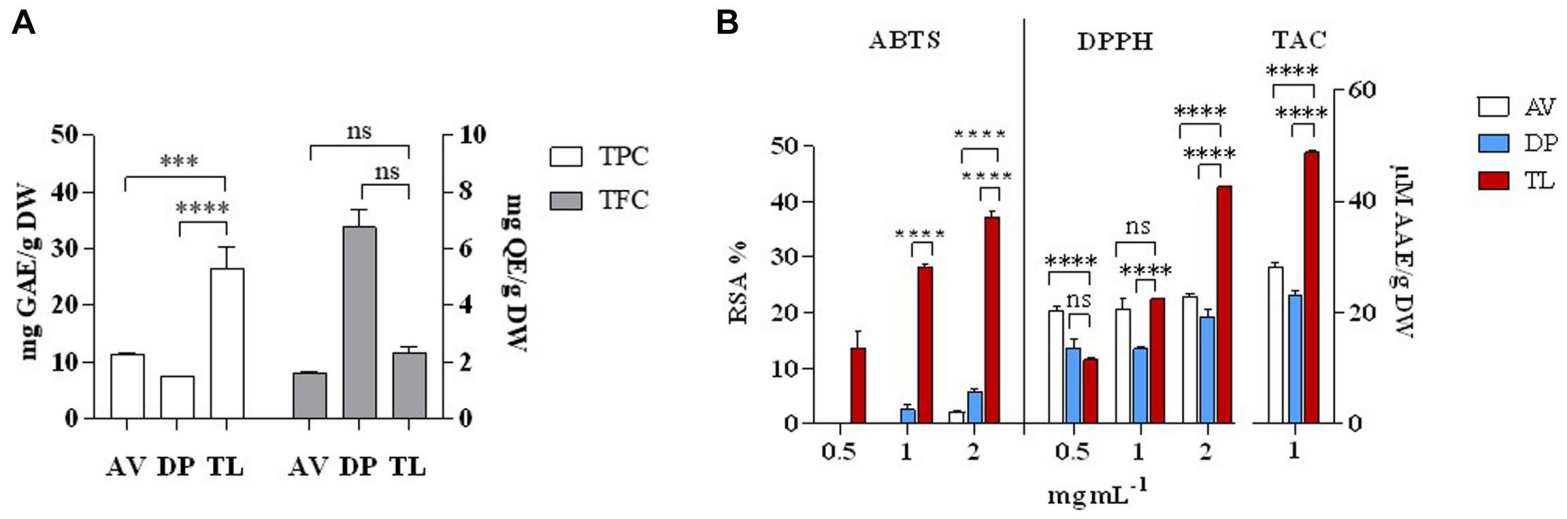
Phytochemical analysis of selected plant species. TPC and TFC **(A)**, ABTS RSA %, DPPH RSA %, and TAC **(B)**. AV—*Adhatoda vasica*, DP—*Diplocyclos palmatus*, and TL—*Thespesia lampas*. ^***^p < 0.001, ^****^p < 0.0001.

Phytochemical analysis of the stem of medicinal plants such as *A. vasica, D. palmatus,* and *T. lampas* showed that *T. lampas* expressed significant levels of TPC, TFC, ABTS, DPPH RSA %, and TAC (*p* < 0.05; Figures 1A,B). TPC measured in the stem were 7.50 (*D. palmatus*), 11.25 (*A. vasica*), and 26.50 mg GAE/g DW (*T. lampas*) whereas TFC measured were 1.57 (*A. vasica*), 2.31 (*T. lampas*), and 6.74 mg QE/g DW (*D. palmatus*). ABTS and DPPH RSA % were found to be significantly higher in *T. lampas* compared with *A. vasica* and *D. palmatus* (*p* < 0.05) in a concentration-dependent manner. ABTS and DPPH RSA % for *T. lampas* ranged from 13.62% to 37.31% and 11.52% to 42.69%, respectively. No ABTS RSA % was detected in lower concentrations of *A. vasica* (0.5 and 1 mg mL^−1^) and *D. palmatus* (0.5 mg mL^−1^). Higher TAC was recorded in the stem of *T. lampas* than in *A. vasica* and *D. palmatus* (48.84, 28.25, and 23.09 μM AAE/g DW, respectively). The values of phenolics and antioxidants of *T. lampas*, *A. vasica,* and *D. palmatus* were similar to the reported studies (Kumaraswamy and Satish, 2008; Dutta and Maharia, 2012; Attar and Ghane, 2017; Shukla et al., 2017). The phenolic and antioxidants of *T. lampas* were detected remarkably higher than *A. vasica* and *D. palmatus*. Polyphenolics are known for their potential role in biological activities as scavengers of free radicals, holding antioxidant capacity (Bhatt et al., 2017; Benabderrahim et al., 2019).

### 3.2 Synthesis, characterization, and mechanism of TSAgNPs and TSAuNPs

**Figure 2:**
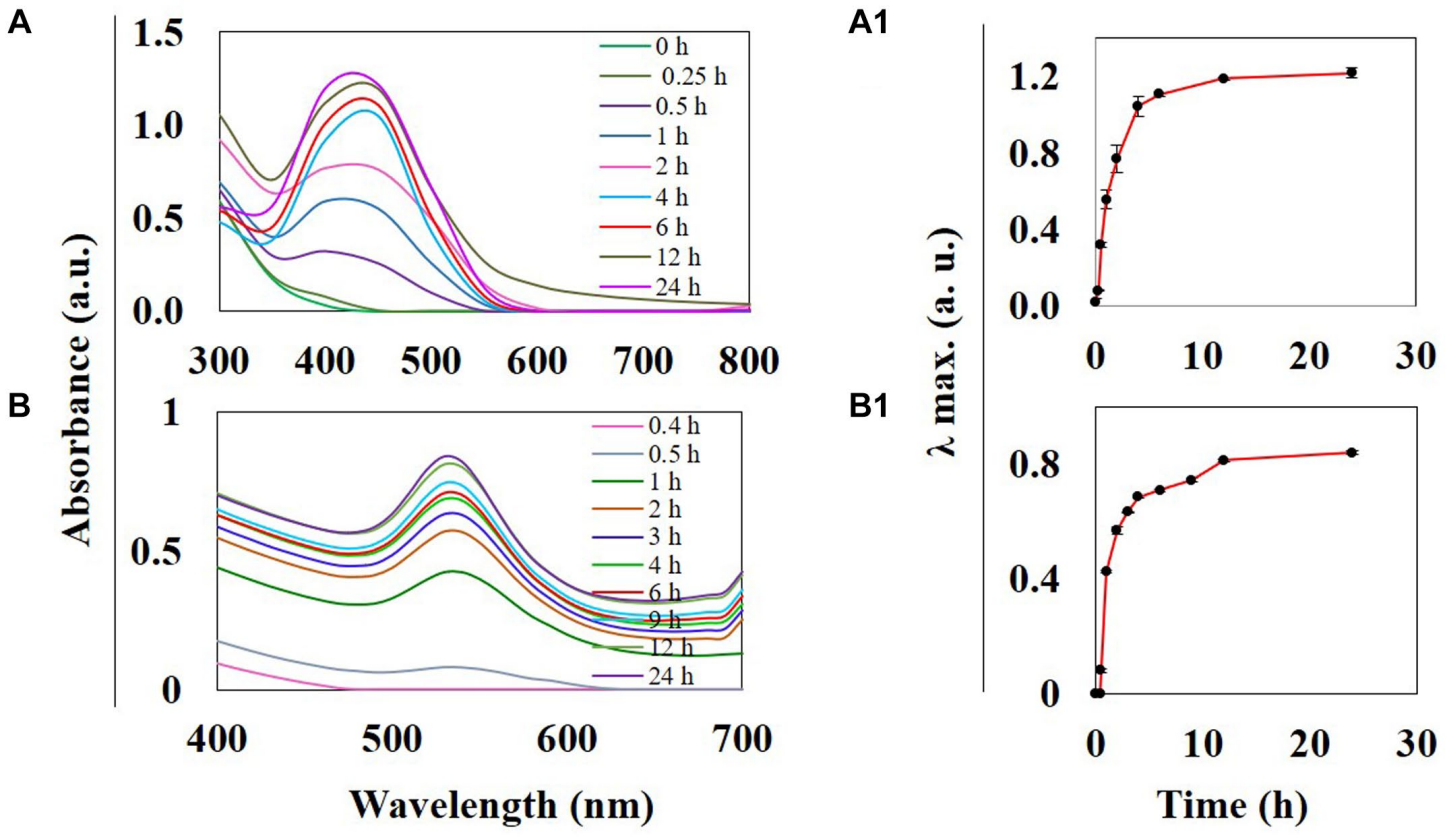
UV–visible spectra of phyto-synthesized TSAgNPs and TSAuNPs were recorded as a function of time. TSAgNPs and TSAuNPs show absorbance at 420 and 530 nm, respectively **(A,B)**. Variation of the corresponding λ_max_ vs. reaction time shows the maximum formation of TSAgNPs and TSAuNPs within 12 h **(A**_**1,**_**B**_**1**_**)**.

*Thespesia lampas* stem extract, when mixed with AgNO_3_ and HAuCl_4_ solutions separately at RT, the colorless AgNO_3_ changed into amber, and the yellow HAuCl_4_ turned into a ruby red-pink color. The instant color change of aqueous AgNO_3_ and HauCl_4_ was observed due to surface plasmon resonance (SPR) excitation (Ramteke C. et al., 2013; Mata et al., 2016; Castillo-Henríquez et al., 2020; Nguyen et al., 2020). The addition of Saq to precursor solutions AgNO_3_ and HauCl_4_ initiated the reduction in Ag^+^ to Ag^0^, causing the synthesis of TSAgNPs and TSAuNPs, respectively (Saxena et al., 2012). Synthesized TSAgNPs and TSAuNPs were monitored at regular time intervals through UV–VIS spectroscopy. The observed absorption bands peaked at 420 and 530 nm for TSAgNPs and TSAuNPs, respectively, and increased steadily with time (Figures 2A,B). Spectra were periodically monitored as a function of time for 24 h. The synthesis of both NPs was completed within 12 h of reaction as λ max approached the plateau with time (Figures 2A_1_,B_1_). A single symmetric absorption peak indicated characteristic SPR of spherical TSAgNPs and TSAuNPs, which are similar to earlier reports (Dauthal and Mukhopadhyay, 2013; Ramteke P. W. et al., 2013; Sre et al., 2015; Sathishkumar et al., 2016).

**Figure 3:**
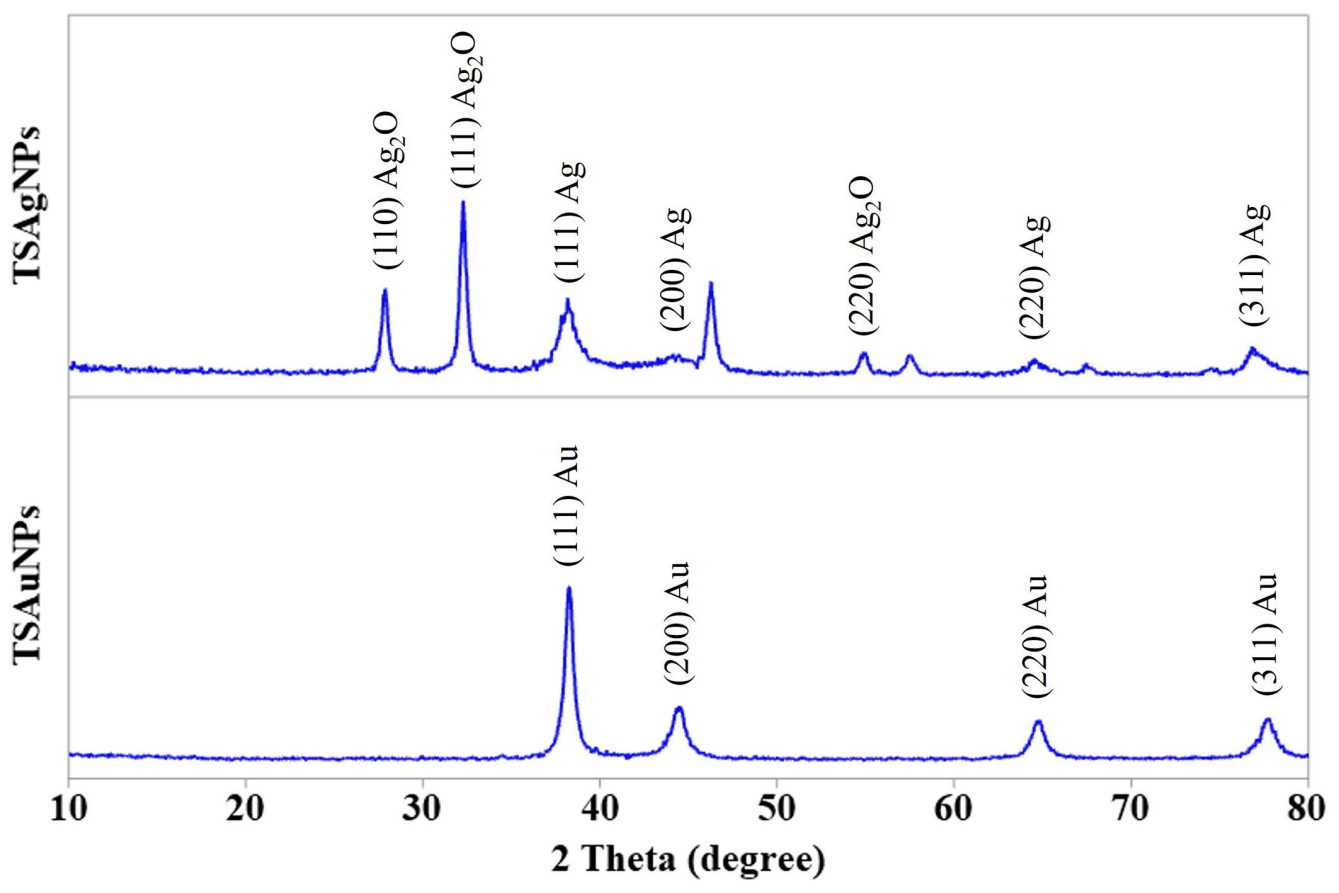
XRD pattern of TSAgNPs and TSAuNPs.

The XRD analysis was carried out to measure the peak intensity, position, width, and size of the crystal. The characteristic diffraction peaks in XRD analysis obtained for TSAgNPs at 38.29, 44.05, 64.38, and 77.36 and TSAuNPs at 38.16, 44.47, 64.78, and 77.79 were plotted in 2*θ* range of 10°–80°, as shown in Figure 3. The diffraction peaks for TSAgNPs and TSAuNPs were indexed to (111), (200), (220), and (311) sets of Bragg’s reflections of crystallite face-centered cubic (fcc) structure. The peaks observed at 27.82, 32.21, and 54.89 were indexed to (110), (111), and (220) planes, which might correspond to the presence of silver oxide NPs (Dhoondia and Chakraborty, 2012; Pawar et al., 2016; Manikandan et al., 2017; Fowsiya and Madhumitha, 2019). The lattice planes were in agreement with the Joint Committee on Powder Diffraction Standards file no. 04–0783 for AgNPs and 04–0784 AuNPs (Philip, 2010; Bindhu and Umadevi, 2013). The mean crystal sizes of TSAgNPs and TSAuNPs were calculated from the full width half maximum using the Debye–Scherrer equation (Philip, 2010).

d=0.9λ/βcosθ

where d is the mean diameter of NPs, λ = 1.5406 = 1.5406, Å is the wavelength of the X-ray source, β is the angular full width half maximum (FWHM) of the peak in radians, and θ is the Bragg angle. The mean crystal sizes of TSAgNPs and TSAuNPs obtained were 13 and 26 nm, respectively. High concentrations of metal ions decreased the peak height and caused the broadening, which indicated that the particles were in the nano range (Bindhu and Umadevi, 2013). The unassigned peaks in the XRD spectrum may be attributed to the crystallization of the phyto-organic phase on the surface of the crystalline nano-sliver (Philip, 2009).

**Figure 4:**
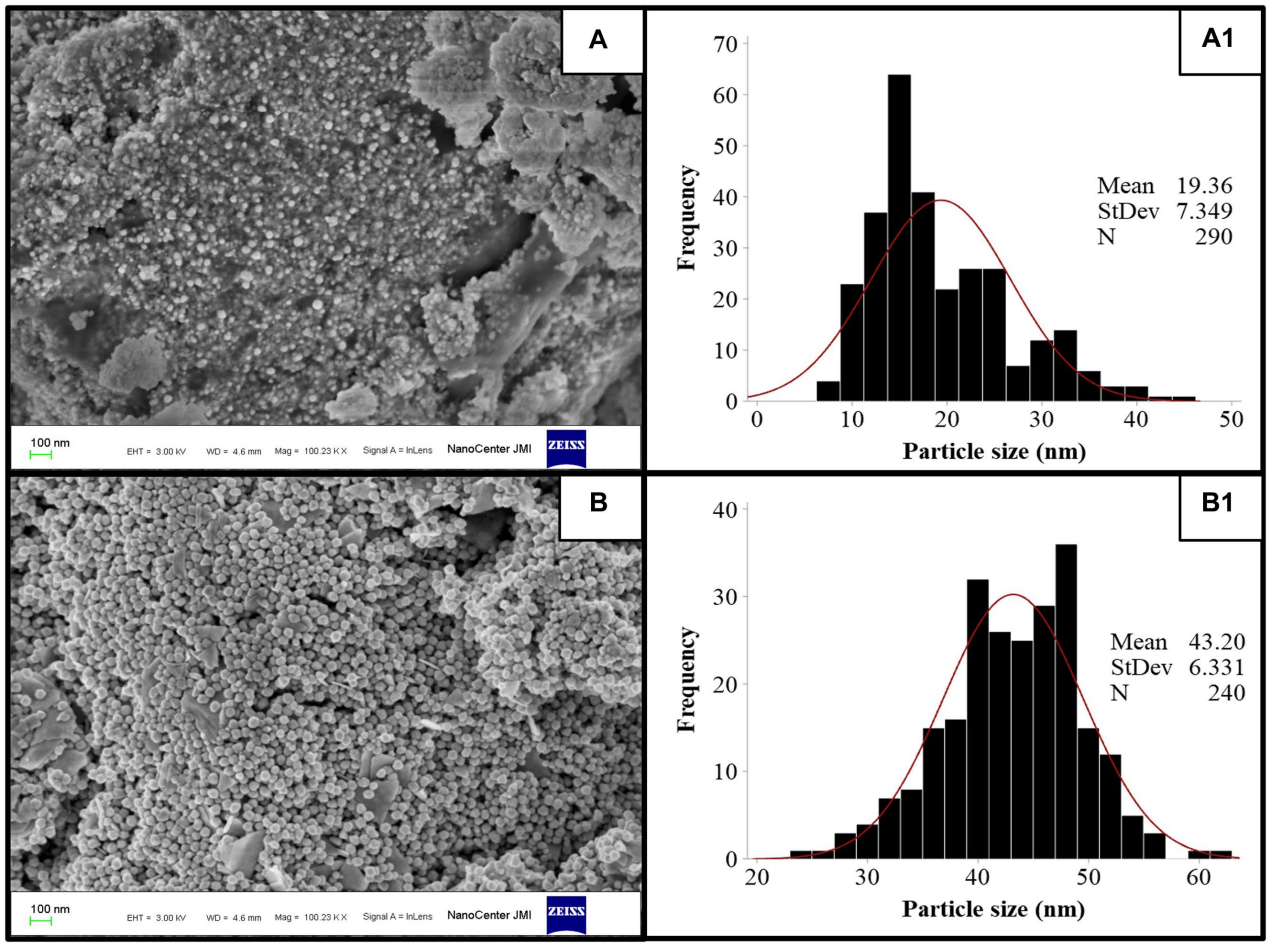
FE-SEM images of TSAgNPs and TSAuNPs **(A,B)** and the corresponding histogram displaying particle size distribution **(A**_**1**_**,B**_**1**_**)**.

**Figure 5:**
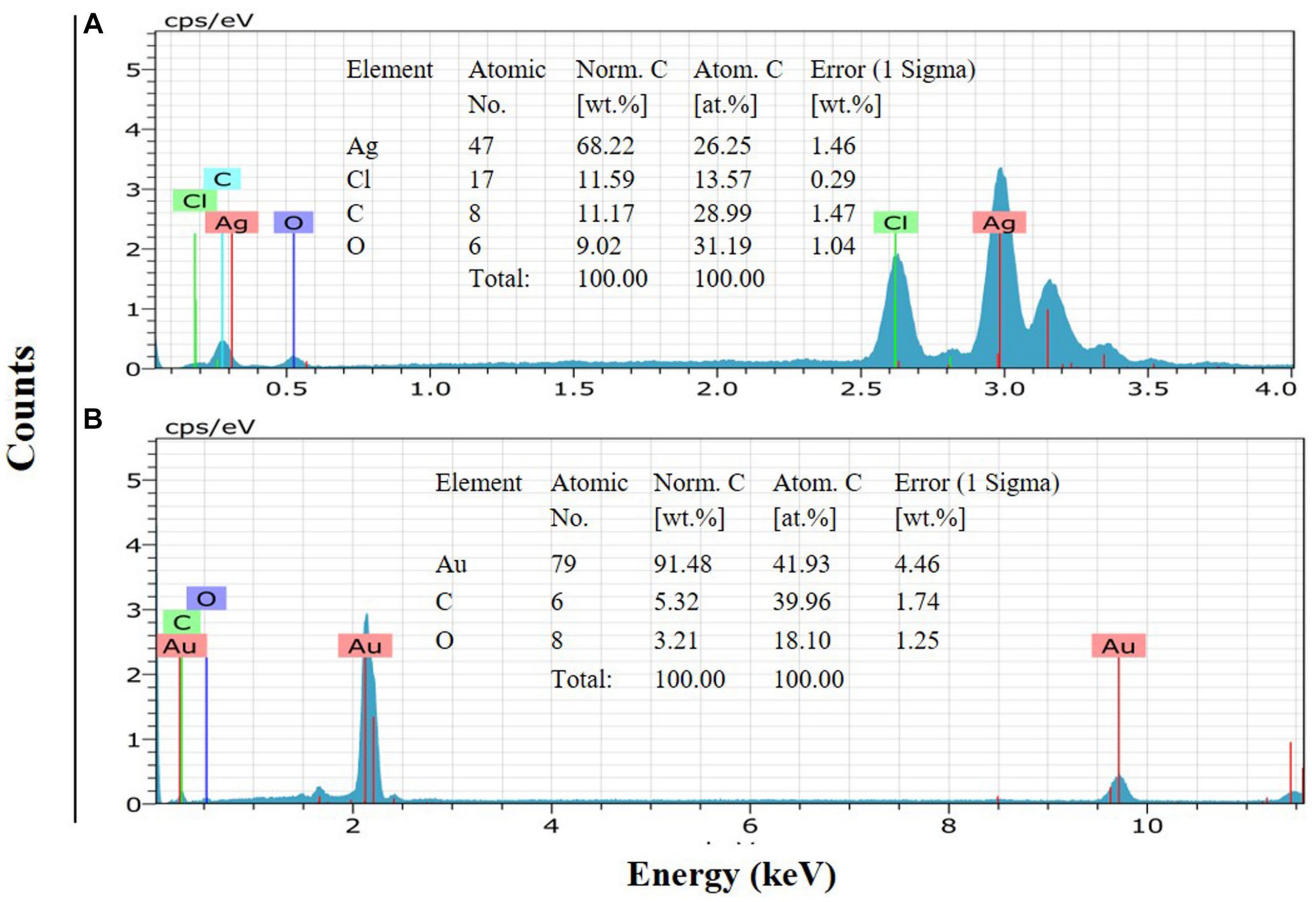
EDS of TSAgNPs and TSAuNPs indicate the elemental presence of silver and gold **(A,B)**.

FE-SEM imaging at 100 nm magnifications revealed the surface morphology and shape of TSAgNPs and TSAuNPs (Figures 4A,B). A narrow diametric size distribution of NPs, indicating polydispersity in the range of 6–46 nm for TSAgNPs (*N* = 290) and 23–63 nm for TSAuNPs (*N* = 240), was realized through the corresponding histograms (Figures 4A_1_,B_1_). The obtained TSAgNPs and TSAuNPs were polydisperse and spherical in shape. The mean diametric sizes of TSAgNPs and TSAuNPs were found to be 19 ± 7.3 and 43 ± 6.3 nm, respectively. EDS analysis confirmed the qualitative and quantitative presence of elemental silver and gold in TSAgNPs and TSAuNPs. Characteristic strong peaks of silver and gold were displayed at 3 and 2 KeV, respectively (Figures 5A,B). The oxygen signal indicated the possibility of silver oxide NPs. The weight percentages of silver and oxygen were 68.22% and 9.02% in TSAgNPs, and the weight percentage of gold was 91.48% in TSAuNPs (Jemal et al., 2017).

**Figure 6:**
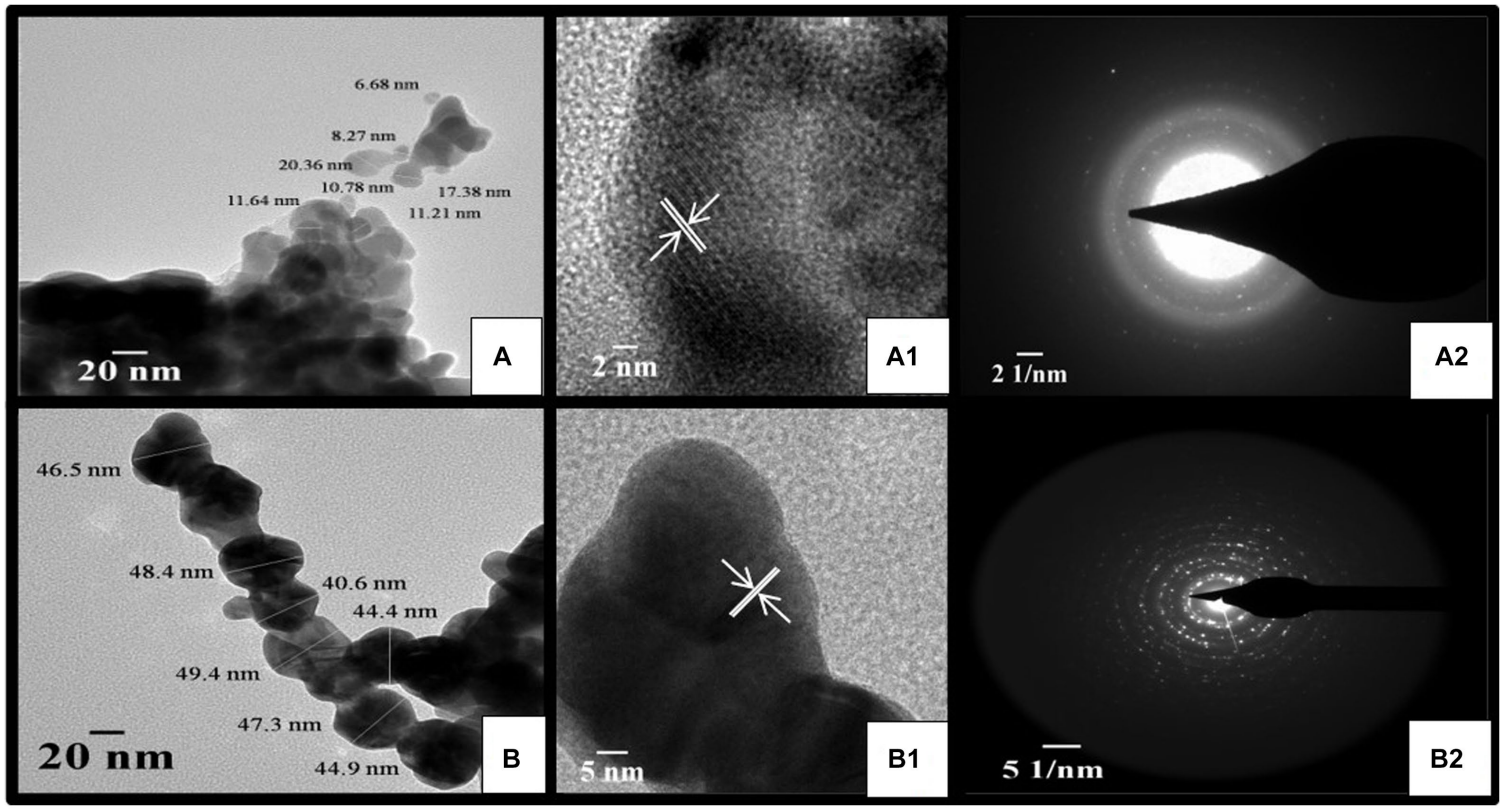
HR-TEM micrograph of TSAgNPs and TSAuNPs **(A,B)**, corresponding lattice fringes with d-spacing of single nanocrystal **(A**_**1**_**,B**_**1**_**)**, and SAED pattern **(A**_**2**_**,B**_**2**_**)**.

TSAgNPs and TSAuNPs observed in HR-TEM micrographs revealed predominantly spherical shape, uniform contrast, polydisperse, and agglomeration forming irregular contours (Figures 6A,B). Uniform contrast reflection in particles indicated the consistency of homogeneous electron density within the volume (Kumari et al., 2015). The mean particle sizes of TSAgNPs and TSAuNPs were obtained to be 12 ± 4.8 and 45 ± 2.9 nm, respectively and were comparable with the results of the SEM polydispersity range (Lokina et al., 2014; Raj et al., 2018). A clear lattice fringe space was measured to be 0.23 nm in each NP, corresponding to the spacing between (111) Bragg’s reflection plane of nanocrystals (Figures 6A_1_,B_1_). The crystalline nature of the lattice space was further evidenced by a selected area electron diffraction (SAED) pattern with bright circular dots of metallic NPs, which corresponded to (111), (200), (220), and (311) planes of crystallite fcc structure (Figures 6A_2_,B_2_). SAED pattern indicated the enhanced growth of crystals, sharing identical orientation (Radziuk et al., 2010). The results obtained in TEM were in agreement with earlier reports (Dauthal and Mukhopadhyay, 2012; Ramteke P. W. et al., 2013). Some layer-kind outside coating was observed on the surface of NPs at high magnification, probably due to the presence of bio-capping of phytochemical moieties from *T. lampas* Saq (Saxena et al., 2012).

**Figure 7:**
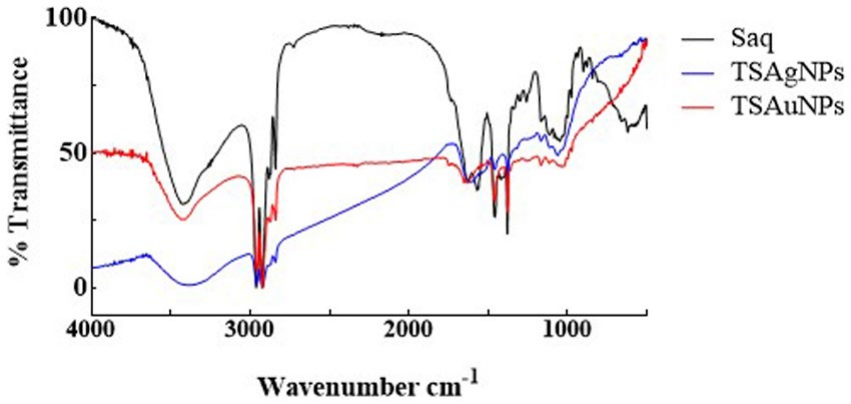
FTIR spectra of Saq, TSAgNPs, and TSAuNPs showing the presence of functional groups.

FTIR spectroscopy was carried out to identify the possible bio-functional groups present in the stem of *T. lampas* which were involved in NP synthesis (Figure 7). Peaks at 3426.72 cm^−1^ in Saq assigned to O-H stretching vibration modes of polyphenolic components shifted to 3385.44 cm-1 in TSAgNPs. The peak at 1567.74 cm-1 in Saq corresponded to amide II and shifted to 1607.25 cm-1 in TSAgNPs (Dauthal and Mukhopadhyay, 2012; Ran et al., 2019). The peak at 1046.90 cm-1 in Saq occurred due to the C-N stretching of aliphatic amines confined to TSAgNPs. The IR spectrum of TSAuNps showed alteration only in a single peak, and the disappearance was observed at 1567.74 cm^−1^. However, other peak positions remain unchanged. The presence of polyphenols, including ellagic acid, tannic acid, quercetin, gallic acid, and rutin, has been reported in *T. lampas* which might be involved during the bioreduction process (Ambrose et al., 2012).

**Figure 8:**
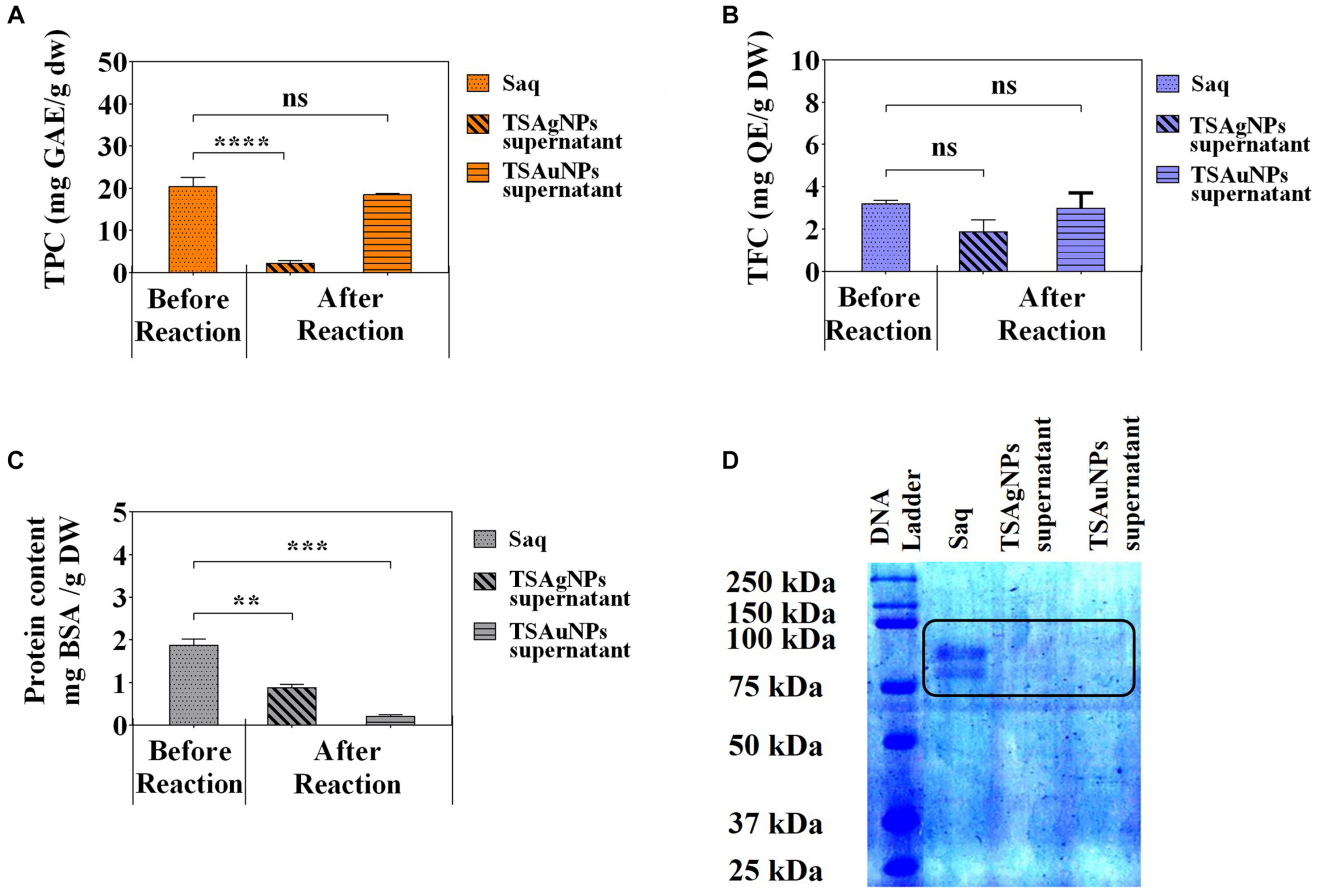
Quantitative phytochemical analysis of Saq, TSAgNPs, and TSAuNPs. Total phenolic content **(A)**, Total flavonoid content **(B)**, protein content **(C)**, SDS-PAGE **(D)**. ^**^p < 0.01, ^***^p < 0.001, ^****^p < 0.0001, ns, non-significant.

**Figure 9:**
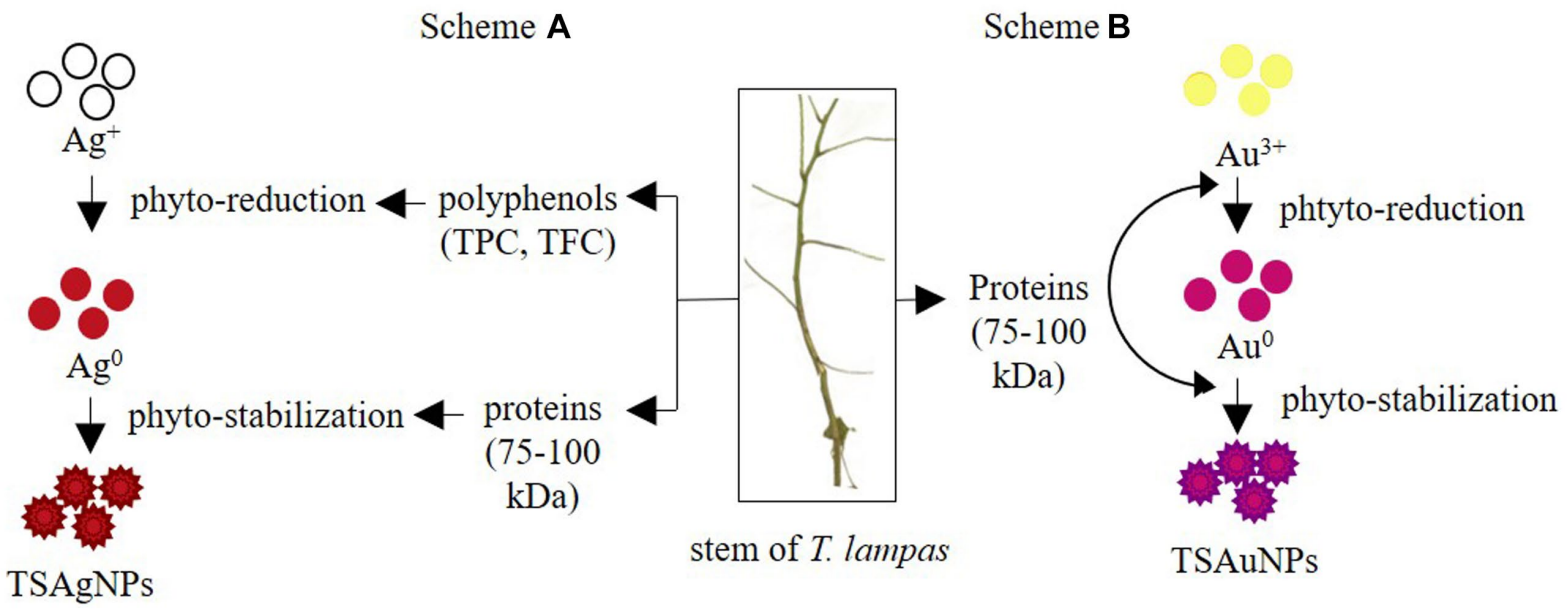
Proposed mechanism for the synthesis of TSAgNPs, and TSAuNPs. Scheme A represented polyphenol and protein acting as bio-reductants and stabilizers for TSAgNPs, and Scheme B represented protein performing dual action of bio-reductant and stabilizer for TSAuNPs.

To identify the contribution of polyphenols and proteins to the synthesis of NPs, TPC, TFC, and total protein were estimated before the synthesis reaction, i.e., Saq and after the synthesis reaction (supernatant left after harvesting NPs; Figure 8). The polyphenol level before the synthesis reaction, i.e., Saq, was noted to decrease in the after synthesis supernatant in TSAgNPs (TPC *p < 0.0001*, TFC *ns*) but not in TSAuNPs (Figures 8A,B). On the other hand, the levels of protein decreased in the after synthesis supernatant of both TSAgNPs (*p < 0.01*) and TSAuNPs (*p < 0.001*; Figure 8C). The involvement of protein in NP synthesis was further shown through SDS-PAGE electrophoresis. Lanes 3 and 4 were loaded with the supernatants of TSAgNPs and TSAuNPs, respectively. Saq illustrated two bands between 75 and 100 kDa as stabilizing proteins that play a crucial role in checking the oxidation of Ag(0) into Ag^+^ (Rodrigues et al., 2013; Chowdhury et al., 2014; Pallavi et al., 2022). The band range of 75–100 kDa evidently disappeared in the after synthesis supernatant of both TSAgNPs and TSAuNPs (Figure 8D). A decrease in polyphenols and protein levels and the disappearance of protein bands in the after synthesis supernatants suggested their possible utilization as bio-reductants and stabilizers that might be absorbed on the surface of NPs during synthesis reactions. Hence, the original forms of these metabolites were probably modified, and consequently, their levels decreased in the after synthesis supernatants (Mishra et al., 2013a; Zheng et al., 2013). Based on the above explanations, two schemes for NP synthesis mechanisms have been proposed (Figure 9). In scheme A, polyphenols acted as bio-reductants for Ag^+^ to form Ag(0), and further protein-aided stabilization of Ag(0) occurred to form phytochemical encapsulated TSAgNPs (Saxena et al., 2012; Mathur, 2014). In scheme B, protein has been suggested to perform a dual function of bio-reductant and stabilizer in synthesizing TSAuNPs (Dauthal and Mukhopadhyay, 2012; Sathishkumar et al., 2016).

### 3.3 Antioxidant properties

**Figure 10:**
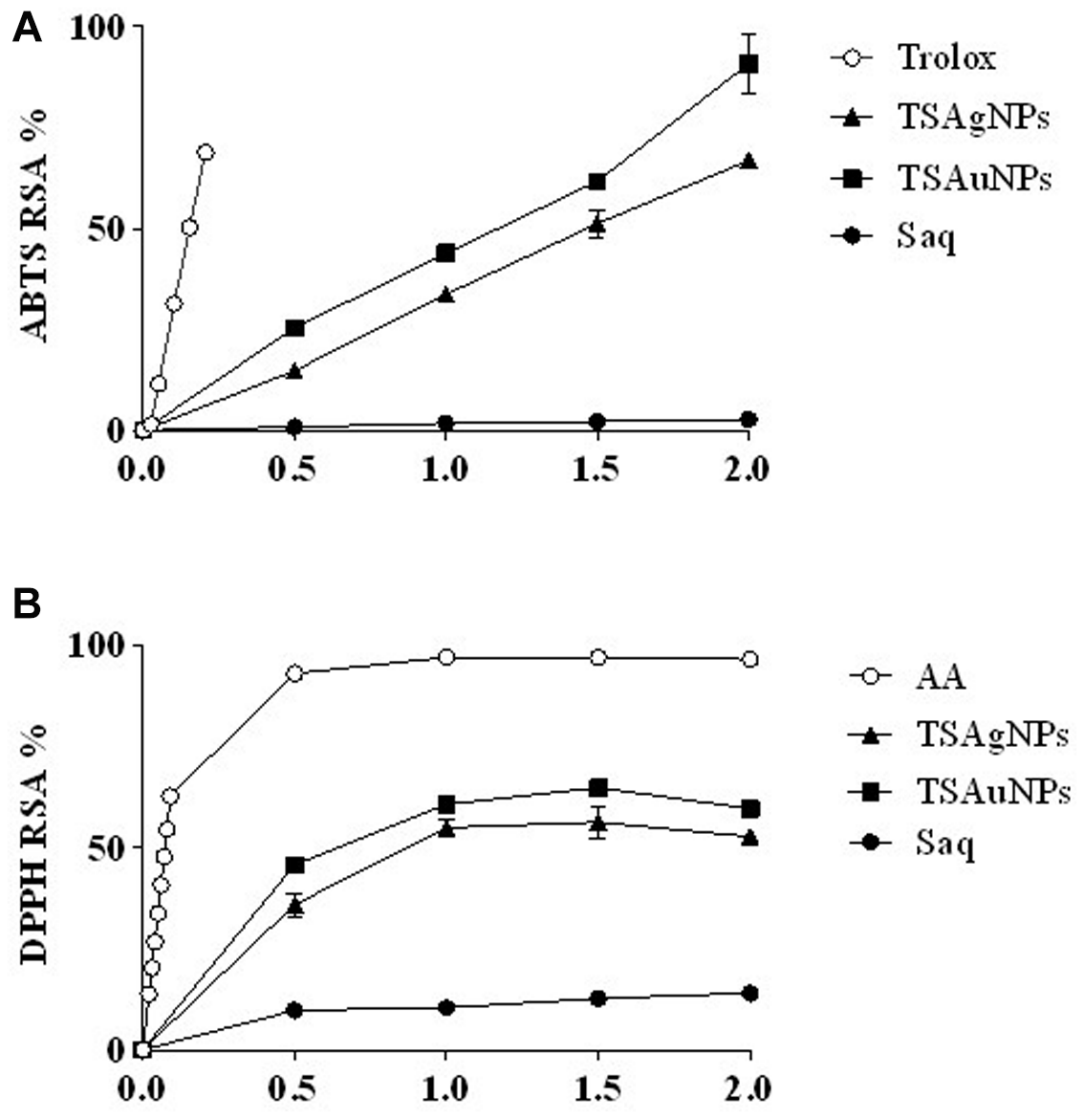
Antioxidant potential of extract and NPs. ABTS RSA % **(A)**, DPPH RSA % **(B)**.

ABTS RSA % of TSAgNPs and TSAuNPs ranged from 14.79 to 66.84% and 25.42 to 90.82%, respectively (Figure 10A). TSAgNPs (EC_50_ = 1.49 mg mL^−1^) and TSAuNPs (EC_50_ = 1.13 mg mL^−1^) exhibited higher ABTS RSA % than Saq (EC_50_ = 36.23 mg mL^−1^) in a concentration-dependent manner. Similarly, DPPH RSA % for TSAgNPs (EC_50_ = 0.88 mg mL^−1^) and TSAuNPs (EC_50_ = 0.65 mg mL^−1^) was found higher than Saq (EC_50_ = 14.00 mg mL^−1^; Figure 10B). TSAuNPs were found to be better scavengers of free radicals than TSAgNPs. The improved radical quenching ability of NPs may be attributed to the (i) electron transfer property that neutralized the free DPPH and ABTS radicals and (ii) intrinsic higher surface-to-volume ratio of NPs, facilitating more linkages between antioxidants and radicals (Dauthal and Mukhopadhyay, 2013; Sathishkumar et al., 2016).

### 3.4 DNA damage protective activity

**Figure 11:**
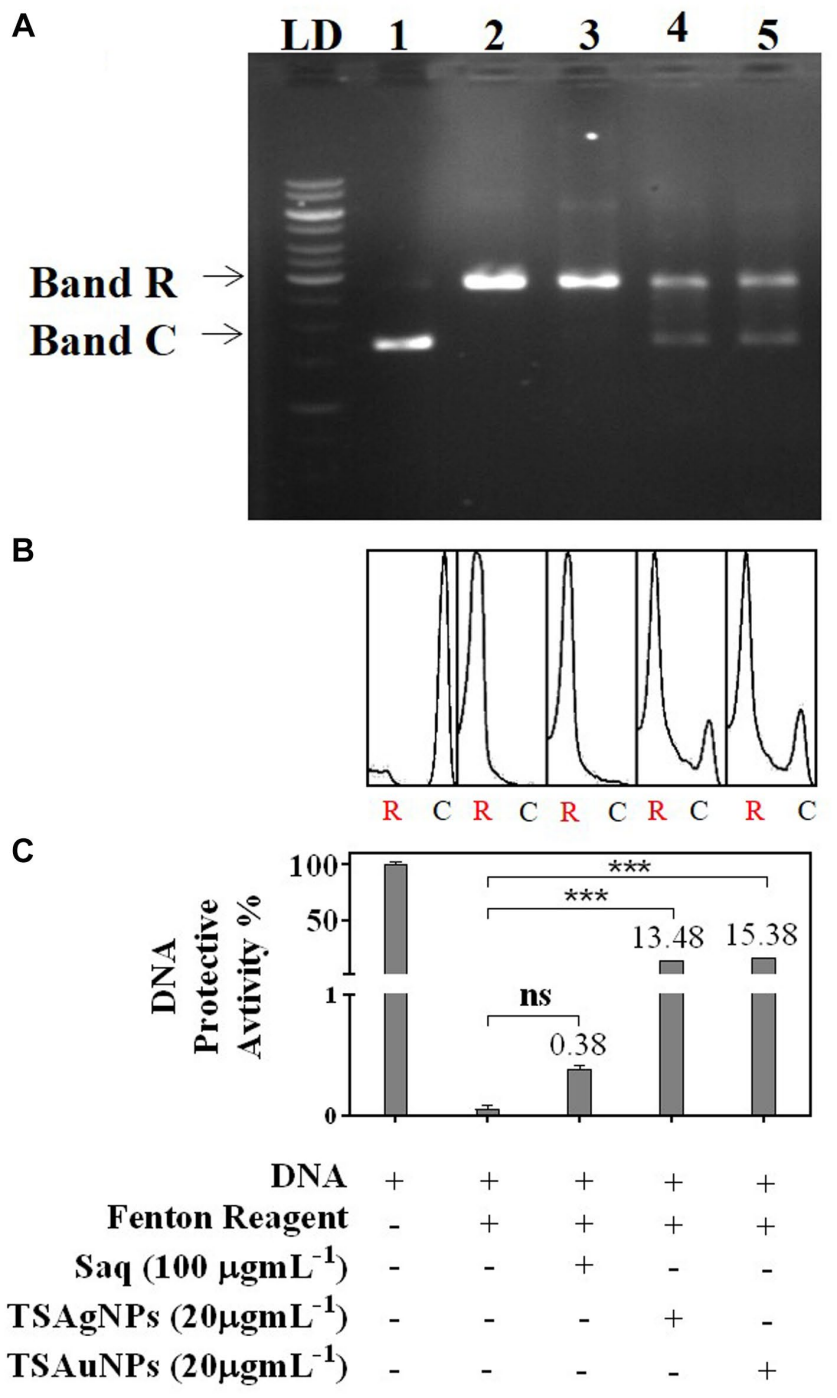
DNA protective activity of Saq, TSAgNPs, and TSAuNPs against ROS-induced damage, depicted as an electrophoretic pattern **(A)**, densitometry analysis against R-DNA **(B)**, and quantification of C-DNA and R-DNA bands **(C)**. LD—ladder DNA, C—circular DNA, and R—relaxed DNA. Lane 1—DNA, Lane 2—DNA + Fenton reagent, Lane 3—DNA + Fenton reagent + Saq, Lane 4—DNA + Fenton reagent + TSAgNPs, and Lane 5—DNA + Fenton reagent + TSAuNPs. ^***^p < 0.001; ns, non-significant.

In gel electrophoresis, Lane 1 contained reference DNA—a native supercoiled circular form of DNA (C-DNA) denoted by Band C. Lane 2 contained a mixture of DNA and a Fenton reagent (Figure 11A). Hydroxyl radicals generated during Fenton reactions exerted oxidative stress, leading to the nicking of native C-DNA to relaxed-DNA form (R-DNA), shown as B and R in the electrophoretic pattern (Figure 11A; Soumya et al., 2013). Saq, TSAgNPs, and TSAuNPs were mixed along with DNA and Fenton reagent in Lanes 3, 4, and 5, respectively, to assess their ability to protect against nicking of C-DNA to R-DNA. Densitometric analysis was performed to measure band intensity using ImageJ software (Figure 11B). The quantification of the electrophoretic image showed that Saq was poorly protective (0.38%) toward DNA nicking, and thus, intense B and R of relaxed DNA were observed in the electrophoretic pattern (Figure 11C). However, TSAgNPs and TSAuNPs were able to recover 13.48% and 15.38% of C-DNA significantly (*p* < 0.001) from hydroxyl damage and aid DNA in retaining its native form observed as light B and C in Lanes 4 and 5. The electron accepting/donating property of NPs led to the interconversion of Ag(0)/Au(0) to Ag^+1^/Au^+1^ which may check ferric ion reduction to ferrous and thus interfered with the Fenton reactions (Ramamurthy et al., 2013; Ajitha et al., 2016). The results suggested the therapeutic quality of TSAgNPs and TSAuNPs and their utilization in stress-induced disorders such as diabetes and cancer (Ramamurthy et al., 2013; Soumya et al., 2013).

### 3.5 Antibacterial investigations

**Figure 12:**
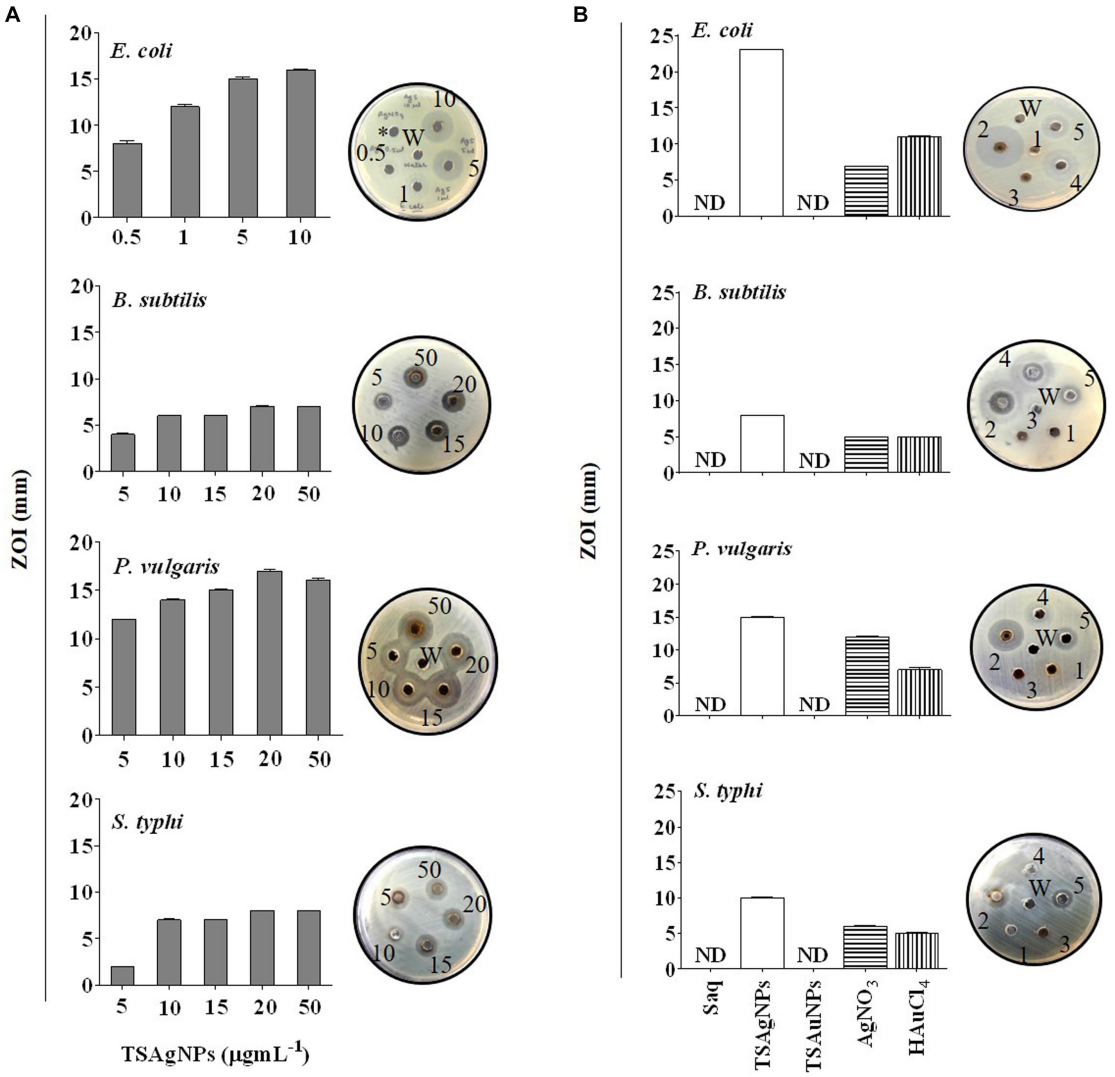
Concentration-dependent antibacterial activity of TSAgNPs (0.5–50 μg mL^−1^) against *E. coli*, *S. typhi*, *B. subtilis*, and *P. vulgaris* with representative bacterial plates **(A)**, comparative antibacterial activity of 50 μL Saq, TSAgNPs and TSAuNPs with representative bacterial plates **(B)**. 1—Saq (5%), 2—TSAgNPs (20 μg mL^−1^), 3—TSAuNPs (20 μg mL^−1^), 4—AgNO_3_ (2 mM), 5—HAuCl_4_ (2 mM), W—water.

The broad-spectrum antibacterial nature of TSAgNPs and TSAuNPs was evaluated in this study. According to the World Health Organization, there are limited antimicrobial agents for gram-negative bacteria for which novel antibiotics are a priority (Al-Ansari et al., 2019). Therefore, three gram-negative (*E. coli*, *P. vulgaris*, and *S. typhi*) and one gram-positive (*B. subtilis*) pathogenic bacteria were included in this study. The antibacterial activity of TSAgNPs and TSAuNPs was performed as i) a concentration-dependent study and ii) a comparative study. The bacterial inhibition of TSAgNPs followed a concentration-dependent (0.5–50 μg mL^−1^) mode of action, where ZOI was observed to increase with increasing concentration. ZOI ranged from 8 ± 0.3 to 16 ± 0.1 for *E. coli*, 4 ± 0.1 to 7 for *B. subtilis*, 12 to 16 ± 0.2 for *P. vulgaris*, and 2 to 8 for *S. typhi* (Figure 12A). However, 20 μg mL^−1^ of TSAgNPs was noted to be effective ZOI, causing maximum inhibition against all pathogens. A comparative antibacterial study among TSAgNPs (20 μg mL^−1^), TSAuNPs (20 μg mL^−1^), Saq (5%), AgNO_3_ (2 mM), 5—HAuCl_4_ (2 mM), and TSAgNPs exhibited significant inhibitory effects against *E. coli* (23 ± 0.0 mm) followed by *P. vulgaris* (15 ± 0.1 mm), *S. typhi* (10 ± 0.0 mm), and *B. subtilis* (8 ± 0.0 mm). TSAgNPs showed inhibition in the order of *E. coli* > *P. vulgaris* > *S. typhi* > *B. subtilis* (Figure 12B). There was no noticeable ZOI formation for treatments of Saq or TSAuNPs. Silver NPs have been reported earlier for effective antibacterial studies in different reports that interfere with the cell membrane permeability, causing cell death (Azam et al., 2012; Dadi et al., 2019). AgNPs were found to be more effective against gram-negative bacteria than gram-positive because the thinner glycan layer in the cell wall makes the former more vulnerable to antibacterial agents (Shrivastava et al., 2007).

### 3.6 Cytotoxicity of TSAgNPs and TSAuNPs on 2D monolayer and 3D spheroid tumor

**Figure 13:**
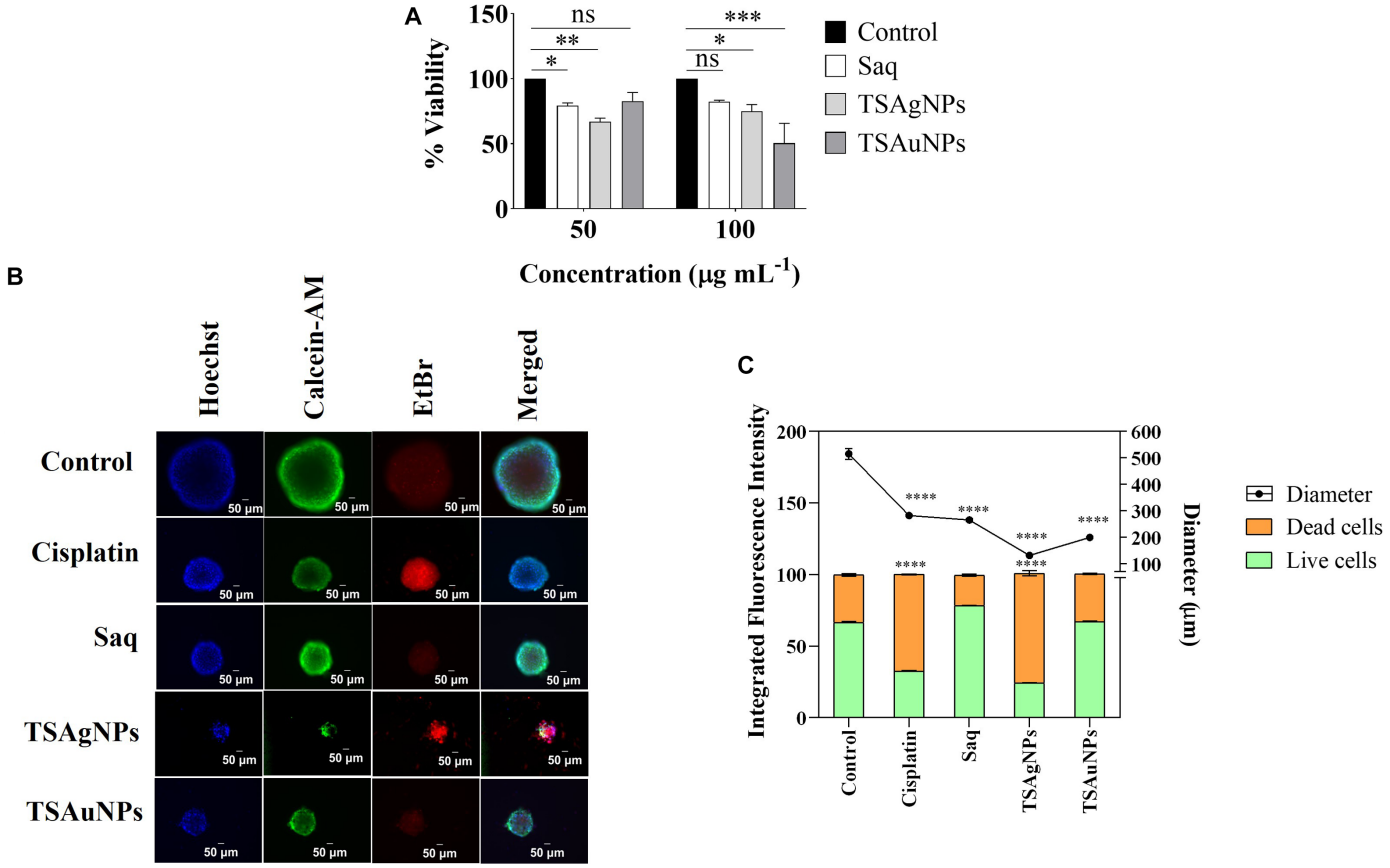
*In vitro* cytotoxicity of Saq, TSAgNPs, and TSAuNPs against FaDu HNSCC. MTT assay for Saq, TSAgNPs, TSAuNPs (each 50 and 100 μg mL^−1^), and Cisplatin (5 μg mL^−1^), after 24 h in 2D condition **(A)**, immunofluorescence images of anti-tumor efficacy for the treatment groups Saq, TSAgNPs, TSAuNPs (each 100 μg mL^−1^), and Cisplatin (10 μg mL^−1^) on FaDu-derived spheroid in 3D condition **(B)**, quantification of the integrated fluorescence intensity % and diametric size of Saq, TSAgNPs, TSAuNPs, and Cisplatin treated spheroid **(C)**. ^*^p < 0.05, ^**^p < 0.005, ^***^p < 0.001, ^****^p < 0.0001, ns, non-significant.

Cancer is a challenging disease in the present healthcare system (Mao et al., 2022). Plant-mediated silver and gold NPs have emerged as robust solutions for several cancer types (Rossi and Blasi, 2022; Chaturvedi et al., 2023). Here, the cytotoxicity of TSAgNPs and TSAuNPs was first validated *in vitro* on the FaDu HNCC 2D monolayer using an MTT cell proliferation assay. In 2D conditions, TSAgNPs reduced the cell viability by 66.97% at 50 μgmL^−1^ (*p* < 0.005) and 74.9% at 100 μg mL^−1^ (*p* < 0.05) as compared with the control (Figure 13A; Barua et al., 2017). NPs at higher concentrations have been reported to interfere with the absorbance wavelength of MTT, resulting in higher absorbance values (Diaz et al., 2008; Ghasemi et al., 2021). TSAuNPs were significantly effective (50.47%) at a higher dose of 100 μg mL^−1^ (*p* < 0.001). The anti-tumor efficacy of TSAgNPs and TSAuNPs was measured through multiple staining on FaDu-derived cancer spheroids that resemble the 3D organization of *in vivo* tumor conditions. Immunofluorescence images of the spheroid with treatment groups are shown in Figure 13B. The Hoechst-stained nucleus of the cancer cell with fluorescent blue signals reflected the live-dead cell population of the spheroid. The AM-stained live cell populations with fluorescent green signals in the peripheral layer and EtBr-stained dead cell populations with fluorescent red signals in the inner necrotic core were measured for integrated fluorescence intensity (IFI) % (Figure 13B). The live cells IFI % for TSAgNPs (100 μg mL^−1^) and Cisplatin (10 μg mL^−1^) significantly reduced to 24.31% and 32.56% compared with the control, respectively (*p* < 0.0001; Figure 13C). The IFI % of dead cells was maximum for TSAgNPs (76.65%), followed by Cisplatin (67.58%). However, TSAuNPs (100 μg mL^−1^) and Saq (100 μg mL^−1^) were found to be ineffective against tumor growth. Additionally, the diameter of the spheroid was measured using Merged fluorescent signals that were critically reduced for all the treatments compared with the control (*p* < 0.0001). The spheroid diameter of control (515.29 μm) reduced to 130.78 μm (25.38%) for TSAgNPs, 199.49 μm (38.71%) for TSAuNPs, 265.24 μm (51.47%) for Saq, and 281.94 μm (54.71%) for Cisplatin. Although TSAuNPs were not effective against tumor viability, they significantly lowered the spheroidal diameter. These findings were similar to the earlier reports of the cytotoxic efficacy of biologically formulated silver and gold NPs on monolayer and spheroids (Henrique et al., 2022). Here, the comparative 2D and 3D cytotoxicity studies suggested that cancer progression was largely inhibited by TSAgNPs, even better than cisplatin, a well-known chemotherapeutic drug. This promising efficacy of TSAgNPs needs to be explored more mechanistically and can be used further in the research advancements of cancer chemotherapeutics.

## 4 Conclusion

The present study discussed the synthesis and biological activities of environmentally safe and economical TSAgNPs and TSAuNPs from the stem of the ethnomedically important medicinal plant *T. lampas*. The higher levels of phytochemicals in *T. lampas* compared to two other medicinal plants *A. vasica* and *D. palmatus*, suggested *T. lampas* as a potential candidate for the study. TSAgNPs and TSAuNPs employed a facile and one-pot aqueous system at RT, eliminating the need for regular hazardous chemicals and external energy sources. Synthesized TSAgNPs and TSAuNPs were of spherical shape in nano-regime. The study proposed a possible mechanism for the synthesis of NPs, involving polyphenols and proteins as bio-reductants and stabilizers. Furthermore, TSAgNPs and TSAuNPs were found to be multi-responsive to a range of biological activities. NPs were able to scavenge ABTS and DPPH radicals, suggesting their antioxidant potential. NPs were capable of protecting DNA against oxidative stress damage. TSAgNPs may serve as active agents to bacterial pathogens in a concentration-dependent mode. TSAgNPs and TSAuNPs showed promising cytotoxic effects against *in vitro* FaDu HNSCC monolayers. Moreover, NPs promoted the inhibition of FaDu-derived HNSCC spheroid by reducing both the viability of live cells and the size of the spheroid. The findings of the study indicated the promising role of synthesized NPs in pharmacological sectors. However, detailed biomedical activities, *in vivo* investigations, and drug delivery challenges are needed to confirm its therapeutic applicability.

## Data availability statement

The original contributions presented in the study are included in the article/supplementary material, further inquiries can be directed to the corresponding authors.

## Ethics statement

Ethical approval was not required for the studies on humans and animals in accordance with the local legislation and institutional requirements because only commercially available established cell lines were used.

## Author contributions

SN: Conceptualization, Data curation, Formal analysis, Investigation, Methodology, Validation, Visualization, Writing – original draft, Writing – review & editing, Resources, Software. RKS: Formal analysis, Investigation, Methodology, Validation, Writing – review & editing, Conceptualization, Data curation, Software. RPS: Project administration, Resources, Visualization, Writing – review & editing, Methodology. MM: Funding acquisition, Resources, Visualization, Writing – review & editing, Project administration, Methodology. BP: Resources, Supervision, Visualization, Writing – review & editing, Project administration, Formal Analysis, Methodology.
